# Vaccination response to protein and carbohydrate antigens in patients with rheumatoid arthritis after rituximab treatment

**DOI:** 10.1186/ar3047

**Published:** 2010-06-08

**Authors:** Maria Rehnberg, Mikael Brisslert, Sylvie Amu, Kiandoht Zendjanchi, Gunilla Håwi, Maria I Bokarewa

**Affiliations:** 1 Department of Rheumatology and Inflammation Research, Sahlgrenska Academy at University of Gothenburg, Guldhedsgatan 10A, Gothenburg, 405 30, Sweden; 2 Rheumatology Clinic, Sahlgrenska University Hospital, Gröna stråket 14, Gothenburg, 413 45, Sweden

## Abstract

**Introduction:**

Rheumatoid arthritis (RA) is frequently complicated with infections. The aim of our study was to evaluate vaccination response in patients with RA after B-cell depletion by using rituximab.

**Methods:**

Influenza (Afluria) and pneumococcal polysaccharides (Pneumo23) vaccines were given 6 months after rituximab (post-RTX group, n = 11) or 6 days before rituximab treatment (pre-RTX group; n = 8). RA patients never exposed to RTX composed the control group (n = 10). Vaccine-specific cellular responses were evaluated on day 6 after vaccination, and vaccine-specific humoral responses, on day 21.

**Results:**

On day 6 after vaccination, formation of influenza-specific B cells was lower in post-RTX group as compared with the pre-RTX group and controls (*P *= 0.04). Polysaccharide-specific B cells were found in 27% to 50%, being equally distributed between the groups. On day 21, the impairment of humoral responses was more pronounced with respect to influenza as compared with the pneumococcal vaccine and affected both IgG and light-chain production. Total absence of influenza-specific IgG production was observed in 55% of the post-RTX group.

**Conclusions:**

RTX compromises cellular and humoral vaccine responses in RA patients. However, repeated RTX treatment or previous anti-tumor necrosis factor (anti-TNF) treatment did not accentuate these defects.

## Introduction

Infections are one of the important causes of death in rheumatoid arthritis (RA) [1-3]. For that reason, RA patients are advised to be vaccinated against influenza and pneumococci [4,5]. Antirheumatic treatment including conventional disease-modifying drugs and TNF inhibitors [6-8] may negatively affect the immunization response. Inhibitor of folate metabolism, methotrexate (MTX), impairs optimal immunization response, whereas the effect of corticosteroids and azathioprine was less pronounced [9,10]. The combination of MTX and TNF inhibitors induces further deterioration of the immunization response [8]. The use of rituximab (RTX), a monoclonal antibody targeting CD20-expressing B cells, is an efficient novel strategy of RA treatment [11]. Preliminary data suggest that RTX treatment may impair the response to the influenza vaccine [11].

In this study, we evaluated the immunization response in RA patients treated with RTX 6 days after immunization and 6 months before immunization. We observed that RTX treatment impairs B-cell functions regarding cellular and humoral responses. RTX-treated patients showed a disrupted production of vaccine-specific κ-light chains in IgG subclass response with respect to protein and polysaccharide antigens, as compared with controls. However, the repeated courses of RTX treatment and distant exposure to TNF inhibitors induced no further impairment of vaccine-specific response.

## Materials and methods

### Patients and vaccination

Twenty-nine RA patients visiting the Rheumatology Clinic at Sahlgrenska University Hospital, Göteborg, were prospectively enrolled in the study between January 2007 and June 2008 (Table 1). One of the patients in the control group was taking oral prednisolone medication, whereas 11 of 19 patients in the RTX-treated groups had prednisolone (daily dose, 2.5 to 10 mg) (Tables 2 and 3). Rituximab (Roche, Basel, Switzerland), 1,000 mg on days 1 and 15, was given intravenously in combination with 2 mg tavegyl and 1 g orally given paracetamol.

**Table 1:** Clinical and demographic parameters of patients with rheumatoid arthritis

Parameter	Post-rituximab (n = 11)	Pre-rituximab (n = 8)	Controls (n = 10)
Vaccination time	6 months after RTX	6 days before RTX	No RTX
B cells (% of mononuclear cells in circulation) (mean ± SD)	2.2 ± 5.2	4.7 ± 4.1	6.1 ± 2.9
Age, years (mean ± SD, range)	60.4 ± 7.8 (45-70)	65.4 ± 11.5 (55-82)	63.6 ± 12.9 (48-95)
Gender, m/f	1/10	1/7	3/7
Disease duration, years (range)	17.3 ± 13.1 (6-33)	8.6 ± 5.5 (3-18)	7.4 ± 4.6 (2-16)
Erosive	10 (91%)	7 (87%)	9 (90%)
RF, positive	11	8	10
Treatment			
MTX, *n *(mg/week, mean ± SD)	10 (17.7 ± 6.3)^a^	7 (18.7 ± 5.4)^b^	10 (18.3 ± 5.6)
Previous anti-TNF, *n*	10	5	2
Previous RTX, *n*	4	1	0
Time after previous RTX, months	30 months (14-48)	24 months	0

MTX, Methotrexate; RF, rheumatoid factor; RTX, rituximab; SD, standard deviation; TNF, tumor necrosis factor.

^a^One patient was receiving azathioprine treatment.

^b^One patient was receiving chlorambucil treatment.

**Table 2:** Detailed information about medications used in the study cohort

Patients	Prednisolone (mg/day)	MTX (mg/week)	Other
Pre-RTX			
1	0	12.5	
2	5	25	
3	7.5	20	
4	6.25	10	
5	10	20	

Post-RTX			
1	5	20	
2	0	20	Cyclosporin A
3	0	25	
4	0	15	
5	5	10	
6	5	25	
7	0	2.5	Azathioprine
8	0	10	
9	2.5	22.5	Cyclosporin A
10	0	20	
11	5	10	

Controls			
1	0	15	Sulfasalazine
2	5	10	
3	0	25	Sulfasalazine
4	0	10	Hydroxychlorokin + cyclosporin A
5	0	20	Hydroxychlorokin
6	0	20	Etanercept
7	0	20	
8	0	25	Hydroxychlorokin
9	0	20	Infliximab
10	0	20	

MTX, methotrexate; RTX, rituximab.

**Table 3:** Humoral response to vaccination on day 21 in RA patients treated with rituximab

	Controls n = 10	Pre-RTX n = 8	Post-RTX n = 11
Influenza vaccine, % increase			
IgM, median (95% CI) responder, *n*	104 (96-130) 3	120 (98-139) 6	105 (88-132) 4
IgG, median (95% CI) responder, *n*	115 (96-191) 6	143 (72-176) 5	109 (85-139)^a ^5
κ-Light chain median (95% CI)	110 (101-145)^a^	126 (98-163)	105 (93-124)
λ-Light chain median (95% CI)	126 (98-175)^a^	147 (94-154)^a^	113 (93-199)^a^

Pneumococci vaccine			
IgM, median (95% CI) responder, *n*	148 (92-541)^a ^8	107 (93-198) 3	105 (77-322) 5
IgG, median (95% CI) responder, *n*	126 (71-213)^a ^7	178 (102-335)^a ^6	107 (88-151)^b ^4
κ-Light chain median (95% CI)	154 (85/218)^a^	124 (97-211)^a^	105 (94-171)
λ-Light chain median (95% CI)	164 (98/571)^a^	227 (111-308)^a^	138 (88-330)^a^

Pre-RTX group, RA patients treated with RTX 6 days after vaccination; Post-RTX group, RA patients treated with RTX 6 months before vaccination; Controls, RA patients never treated with RTX. The levels of vaccine-specific antibodies at day 0 were taken as 100%. On day 21, the mean increase of antibody levels in the control group was 110%. This value indicated the cutoff for vaccination response in the RTX-treated patients. CI, Confidence interval.

^a^Increase of Ig levels on day 21 compared with day 0, *P *< 0.05.

^b^Increase of Ig levels in post-RTX group was lower as compared with pre-RTX group, *P *< 0.027.

All patients were vaccinated with Pneumo23, a preparation containing capsule polysaccharides of 23 pneumococcal serotypes (MSD, Brussels, Belgium), and Afluria, influenza vaccine (ZLB Pharma, Marburg, Germany), administered intramuscularly. The patients were vaccinated 6 months after RTX treatment (post-RTX group, n = 11), or 6 days before RTX treatment (pre-RTX group, n = 8). As a control group, 10 patients never treated with RTX were included. Blood samples were obtained from a cubital vein on day 0, day 6, and day 21 after vaccination.

The ethics committee at the Sahlgrenska University of Hospital approved this study. All patients gave written informed consent for participation in the study.

Detection of vaccine-specific B cells was performed by using the enzyme-linked immunosorbent spot (ELISPOT), as described [12]. The 96-well nitrocellulose filter plate (Millipore, Molsheim, France) coated with 10 μg/ml poly-L-lysine (Sigma, St. Louis, MO) was incubated overnight with Pneumo23 (diluted 1 to 5) or Afluria (diluted 1 to 20). Cells were seeded in 1 × 10^5^, 2 × 10^4^, 4 × 10^3^, and 8 × 10^2 ^cells/well and incubated for 12 hours. Secreted immunoglobulins (Igs) were detected on a single-cell level by using goat IgG anti-human IgG, IgA, and IgM (Sigma). Each spot corresponded to one vaccine-specific B cell enumerated with a microscope and presented as the median number of spot-forming cells (sfc)/10^6 ^lymphocytes, with a confidence interval (CI) of 95%. The sfc > 50 identified vaccination responders. We previously showed that in immunized RA patients, a cutoff > 5 sfc is safe; however, because we used B cell-depleted RA patients, we increased the cutoff level to > 50 sfc [13].

### Detection of vaccine-specific antibody production

The 96-well plates (NuncMaxisorb) were coated with Pneumo23 (diluted 1 to 5) and Afluria (diluted 1 to 20), blocked and incubated overnight with samples diluted 1:300 and 1:1,200. Goat anti-human IgG_1_, IgG_2 _(both Caltag Laboratories, Burlingame, CA), IgG_3_, IgG_4 _(both Sigma), IgM-HRP (Jackson ImmunoResearch Laboratories, West Grove, PA), total IgG, κ light chain (κ-chain)-HRP, lambda light chain (λ-chain)-HRP (all from Dako Immunoglobulins, Glostrup, Denmark) were used, followed by an appropriate conjugate and substrate. The plates were read at 405-nm and 450-nm wavelengths. Samples drawn on days 0 and 21 were analyzed in parallel; the comparison was done at the same sample dilution. The levels of vaccine-specific antibodies at day 0 were taken as 100%. On day 21, the mean increase of antibody levels in the control group was 110%. This value indicated the cutoff level for vaccination response in the RTX-treated patients.

### Statistical analyses

Results are presented as median [CI 95%]. Continuous parameters of the same patient over time were compared by using the paired *t *test. The groups were compared by using a Mann-Whitney test, and *P *values < 0.05 indicated significant differences between the groups. All statistical analysis was performed by using the Prism 5 software (GraphPad Software, San Diego, CA, USA).

## Results

### Evaluation of B-cell numbers in the study groups

At the time of immunization, the numbers of circulating B cells were evaluated by using flow cytometry. The numbers of B cells in percentages is calculated based on CD19^+^CD3^- ^in the mononuclear population. The distribution in the post-RTX, pre-RTX, and controls is shown in Table 1.

### Vaccine-specific cellular responses after RTX treatment

Cells secreting vaccine-specific Ig were enumerated 6 days after vaccination. At this time, the pre-RTX group had not received RTX and was identical to controls. Influenza = specific IgM B cells were prevalent in the pre-RTX group (median sfc/10^6 ^lymphocytes, 55[0 to 1704] and controls (sfc/10^6 ^lymphocytes 30[0 to 3,500]) as compared with the post-RTX group (sfc/10^6 ^lymphocytes, 3[0 to 277]). Significantly decreased levels of influenza-specific IgM-secreting B cells were detected in the post-RTX group versus controls (*P *= 0.046). In addition, when comparing the post-RTX group with the pre-RTX group, significantly lower levels were found (*P *= 0.044). No differences were found between the pre-RTX group and controls. The number of influenza-specific IgG- and IgA-producing cells was similar, irrespective of RTX treatment. Pneumo23-specific IgM-B cells were not found in any group. The number of B cells producing Pneumo23-specific switched Ig was lower (but not significant) as compared with the influenza-specific B cells. No other differences in Pneumo23-specific response were found between the groups.

### Vaccine-specific humoral responses after RTX treatment

The levels of vaccine-specific Ig were compared on days 0 and 21 (Table 3). We did not find any differences in the increased influenza-specific IgM levels between the groups. The levels of Pneumo23-specific IgM were elevated in controls compared with day 0 (*P *< 0.05). No significant increase of Pneumo23-specific IgM was found in the RTX-treated groups.

On day 21, the mean levels of influenza-specific IgG were significantly increased as compared with day 0 in the post-RTX group (Table 3). When comparing influenza-specific IgG levels in the pre-RTX group and controls, no differences could be detected. A significant increase of Pneumo23-specific IgG was observed in pre-RTX group (*P *= 0.05) and controls (*P *= 0.037), whereas P23-specific IgG production in the post-RTX group was less pronounced. Pneumo23-specific IgG production was significantly higher in the pre-RTX group as compared with the post-RTX group (*P *= 0.027).

The post-RTX group was divided into patients treated with RTX once (n = 7) or repeatedly RTX treated (twice or more; n = 4), and the levels of vaccine-specific IgM and IgG were compared. We observed no differences in the levels of vaccine-specific Ig between the patients treated with RTX once as compared with those who were treated more than once, suggesting that repeated courses of RTX induced no further impairment of the vaccine-specific response.

Furthermore, dividing all RTX-treated patients into those previously treated with TNF-inhibitors (n = 15) and patients not treated with TNF-inhibitors (n = 4) revealed no differences between these groups, suggesting that previous treatment with TNF inhibitors does not affect our results.

### IgG isotypes of vaccine-specific response to influenza and Pneumo23

**Figure 1:**
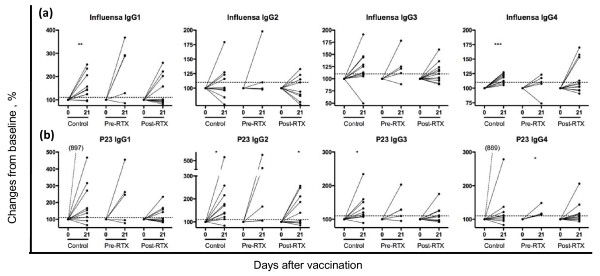
**Changes in IgG subclasses after protein and polysaccharide vaccination in rheumatoid arthritis (RA) patients before and after RTX treatment**. **(a) **Changes at day 21 as compared with day 0 in immunoglobulin G (IgG)_1-4 _are shown after influenza vaccination. **(b) **Changes at day 21 as compared with day 0 in IgG_1-4 _after P23 vaccination. In all figure parts, day 0 levels are set at 100%, and day 21 indicates changes relative to day 0. Dotted line indicates cutoff, set at 110%. Each dot corresponds to one patient, and statistical evaluation was performed by using the paired *t *test; statistical significance is set at *P *< 0.05. *P < 0.05; **P < 0.01; ***P < 0.0001.

The subclass analysis of IgG production showed that the control group produced IgG_1 _and IgG_4 _influenza-specific IgG (Figure 1). In contrast, when combining the RTX-treated patients into one group, no subclass distinction in IgG production could be detected. However, a substantial part of these RTX-treated patients (five of 16; 31%) had no increase of any influenza-specific IgG subclass on day 21 after vaccination (Table 4). Patients in the post-RTX group had the lowest proportion of responders to influenza of any IgG subclass (IgG_1, _four of 11; IgG_2_, six of 11; IgG_3_, six of 11; and IgG_4_, four of 11) (Table 4). Interestingly, four patients in the post-RTX group did not respond in any IgG subclass against influenza immunization. In contrast, only one patient in the pre-RTX group and no one in the control group displayed a total lack of IgG-subclass increase (Table 4).

**Table 4:** Individual responses of total IgG or IgG subclasses after influenza and pneumococcal polysaccharide immunization

Patients	Total IgG	IgG_1_	IgG_2_	IgG_3_	IgG_4_	Total IgG	IgG_1_	IgG_2_	IgG_3_	IgG_4_	Previous RTX
Influenza						P23					

Pre-RTX											
1	**176**	**289**	99	**125**	**117**	**152**	95	105	**110**	**115**	1
2 ^a^	94	86	98	89	74	**102**	**454**	**534**	**203**	**117**	no
3 ^a^	**163**	**128**	**110**	**112**	**111**	**204**	**262**	107	95	**115**	no
4 ^a^	**154**	**292**	**198**	**178**	**108**	**335**	77	**167**	**129**	**148**	no
5 ^a^	**132**	**368**	99	**121**	**123**	**217**	**246**	**458**	**128**	**113**	no

Post-RTX											
1^a^	**139**	**202**	**123**	**116**	**153**	**151**	83	85	95	102	no
2	**126**	**260**	**133**	**136**	**158**	**110**	103	104	95	101	2
3	109	93	**115**	**110**	**107**	107	**143**	105	107	**116**	no
4	100	97	89	102	91	102	104	98	**111**	**112**	no
5^a^	**115**	89	72	100	96	93	87	**211**	**112**	108	no
6^a^	85	93	76	87	102	88	82	**187**	96	107	3
7	98	82	94	96	97	107	95	94	98	94	no
8	103	99	86	**124**	**113**	95	**233**	**257**	**126**	**117**	no
9^a^	104	102	**115**	91	103	**126**	92	105	92	98	1
10	**127**	**222**	**115**	**160**	**170**	104	**159**	**248**	**175**	**206**	2
11^a^	**134**	**157**	**110**	**119**	**109**	**122**	**167**	**140**	**125**	**144**	no

Controls											
1	**117**	95	84	**113**	108	**213**	91	**171**	89	83	no
2^a^	**191**	**252**	96	**125**	**127**	**115**	**897**	**521**	**235**	**278**	no
3	100	**157**	100	50	105	**139**	137	**178**	109	95	no
4	96	**145**	97	105	**113**	**136**	467	**216**	**159**	**137**	no
5	**121**	**205**	101	**191**	**126**	105	**114**	**112**	107	**111**	no
6	**116**	**124**	**123**	108	**119**	97	**154**	**136**	**151**	**112**	no
7	**113**	**123**	**179**	**111**	**111**	71	**316**	**259**	**120**	**122**	no
8	**174**	**234**	**127**	**146**	**121**	**161**	**167**	**140**	108	105	no
9	97	98	72	**144**	**123**	**111**	65	84	**114**	101	no
10	99	**142**	**116**	**129**	**124**	**194**	**271**	**122**	**132**	**889**	no

^a^Prednisolone, 2.5-10 mg/day.

Controls, RA patients never treated with RTX; P23, pneumococcal polysaccharide vaccine 23; pre-RTX group, RA patients treated with RTX 6 days after vaccination; post-RTX group, RA patients treated with RTX 6 months before vaccination; RTX, rituximab.

The levels of vaccine-specific antibodies at day 0 were taken as 100%. On day 21, the mean increase of antibody levels in the control group was 110%. This value indicated the cutoff for vaccination response in the RTX-treated patients.

Bold numbers and gray boxes indicate levels equal to or greater than the cutoff level (110%).

For Pneumo23 vaccination, controls revealed a significant increase of IgG_2 _and IgG_3_, whereas post-RTX patients showed an isolated increase of IgG_2_, and pre-RTX patients, of IgG_4 _(Figure 1). A total lack of IgG-subclass response against P23 was observed in four patients in the post-RTX group, but none in the pre-RTX group or in the control group (Table 4).

A previous course of RTX did not affect the immune responses to influenza or P23, because only one of five patients that did not respond to influenza had a previous course of RTX, and two of four patients that did not respond to P23 had a repeated course of RTX (Table 4). No difference in IgG subclasses was observed in patients with respect to previous treatment with TNF-α inhibitors.

### Vaccine-specific production of Ig light chains after RTX treatment

We measured the development of vaccine-specific light-chains to evaluate functional B-cell populations in RTX-treated patients. On day 21, the levels of influenza-specific Ig containing λ-chains (Ig-λ) were elevated in all groups, irrespective of RTX treatment (Table 3). In contrast, influenza-specific Ig-κ was elevated only in controls (*P *= 0.001), but neither in post-RTX nor in pre-RTX groups, suggesting that RTX may disrupt the production of Ig-κ. The pattern of Pneumo23-specific Ig-κ was similarly elevated in the pre-RTX group (*P *= 0.05) and in controls (*P *= 0.01).

The pattern of light-chain production correlated to vaccine-specific IgG levels. None of six post-RTX patients with the absence of an increase in influenza-specific IgG subclasses developed increase in influenza-specific Ig-| and Ig-| production. Only one of six patients had repeated RTX treatments; thus, no accumulation of light-chain suppression correlated with the number of RTX treatments.

## Discussion

In the present study we investigated the vaccine response to protein (influenza) and polysaccharide (pneumococcal) preparations in RA patients treated with RTX 6 days after vaccination (pre-RTX group) and 6 months before vaccination (post-RTX group). The response was compared with that of RA patients never treated with RTX (controls).

Previous reports on vaccination after RTX treatment showed a detectable but reduced humoral response to influenza in RA patients [11,14,15]. The vaccination time point in those studies was chosen after RTX treatment and corresponded to the post-RTX group in our study. We chose the time point of 6 months after RTX treatment because, at that time, antigen-naïve B cells (CD19^+^IgD^+^CD27^-^) start repopulating the bone marrow and circulation of RA patients [16]. The post-RTX group had measurable amounts of B cells in peripheral blood. Because we did not find any significant differences in frequencies between any of the study groups, we draw the conclusion that the immunization responses detected are not due to different numbers of B cells in our study groups. The vaccination time point corresponding to 6 days before RTX treatment (pre-RTX group) allowed us to study the role of B cells in the early events of the immune response. During the first 6 days after vaccination, the primary antigen-specific B cells are formed. These primary B cells express vaccine-specific Ig on the surface, detected in our study with ELISPOT. However, the primary B cells do not secrete vaccine-specific Ig, because no antibody levels may be detected in the circulation. It was previously shown in our laboratory that antigen-specific B cells are detected in the circulation between days 5 and 7 after vaccination and thereafter migrate to peripheral lymphoid organs [13]. At this phase of vaccine response, B cells were depleted in the pre-RTX group, and humoral (mature) responses were evaluated in these patients on day 21.

We observed that RTX treatment impairs B-cell functions. RTX treatment reduced development of influenza-specific B cells at the initial step of immune response, as long as 6 months after RTX treatment. Notably, this impaired cellular response resulted in a selective reduction of IgM-expressing B cells, whereas the numbers of influenza-specific IgG and IgA cells were not changed in the post-RTX group. Because the numbers of B cells producing IgG and IgA were unaffected, one could suggest that a secondary vaccination response was observed, characterized by the activation of already existing influenza-specific memory B cells that had not been depleted by the RTX treatment. Similar levels of influenza-specific total IgG levels between the RTX-treated groups and controls further support this hypothesis. However, half of the post-RTX patients showed a total absence of vaccine-specific IgG production in all subclasses. Interestingly, the pre-RTX group developed an adequate humoral response both to protein and to polysaccharide antigens. Additionally, RTX-treated patients showed no distinction in IgG-subclass response with respect to protein and polysaccharide antigens present in controls. The influenza-specific response in the controls was characterized by production of IgG_1 _and IgG_4_. Such a distribution between vaccine-specific IgG subclasses was not present in RTX-treated patients, suggesting that RTX disrupted the "normal subclass" pattern of IgG production.

In our previous study, we showed that RTX induces total depletion of the antigen-naïve B-cell population (CD19^+^IgD^+^CD27^-^), both in blood and in bone marrow of RA patients [12]. To investigate further whether RTX selects any B-cell subclass for elimination, we evaluated light-chain production in RTX-treated patients after immunizations. Interestingly, we observed that RTX-treated patients had a disrupted production of vaccine-specific κ-light chains. This finding may be due to either an enhanced sensitivity to RTX of κ-light chain-expressing B cells, resulting in an enrichment of λ-light chain-expressing B cells or a switch from κ-light chain to λ-light chain production.

Another and more speculative and controversial theory is that during RTX treatment, the B cell could switch from κ-light chain to λ-light chain production in the periphery after antigenic challenge. Further investigations are needed to confirm this theory. However, it has been suggested that antigenic challenge, at least in the bone marrow of mice, can cause a switch from κ-light chain to λ-light chain, which indirectly may support our findings [17,18].

Aiming to understand whether previous biologic treatment contributed to the impaired B-cell function, we compared vaccine-specific responses between the patients treated with RTX once and those who were treated repeatedly. We observed no differences in the levels of vaccine-specific Ig between those groups, suggesting that repeated courses of RTX induced no further impairment of the vaccine-specific response. Additionally, the RTX-treated groups included patients previously exposed to and but for whom TNF inhibitors failed. The vaccine-specific response in these patients was similar to that in patients never exposed to TNF inhibitors, suggesting that distant exposure to TNF inhibitors was unlikely to contribute to the observed B-cell dysfunctions. This suggestion is in concordance with previous reports of sufficient humoral response to influenza vaccine in patients treated with TNF-inhibitors [6,10,19].

## Conclusions

In this study, we evaluated the vaccination response in RA patients treated with RTX 6 days after vaccination and 6 months before vaccination. We observed that RTX treatment impairs B-cell functions regarding cellular and humoral responses. RTX-treated patients showed a disrupted production of vaccine-specific κ-light chains and no distinction in IgG-subclass response with respect to protein and polysaccharide antigens present in controls. However, the repeated courses of RTX treatment and distant exposure to TNF inhibitors induced no further impairment of the vaccine-specific response.

## Abbreviations

Ig: immunoglobulin; MTX: methotrexate; RA: rheumatoid arthritis; RTX: rituximab; TNF: tumor necrosis factor.

## Competing interests

The authors declare that they have no competing interests.

## Authors' contributions

MR performed the science, analyzed data, and wrote the article. MB planned the science, performed the science, analysed data, and wrote the article. SA performed the science and analyzed data. KZ collected patient material. GH collected patient material. MIB planned the science, collected patient material, analyzed data, and wrote the article.
